# Reply: Comment on ‘Contribution of pelvic and para-aortic lymphadenectomy with sentinel node biopsy in patients with IB2–IIB cervical cancer’

**DOI:** 10.1038/bjc.2012.226

**Published:** 2012-06-07

**Authors:** E Chéreau, E Darai

**Affiliations:** 1 Department of Gynecology-Obstetrics, Hôpital Tenon, Assistance Publique des Hôpitaux de Paris, CancerEst, Inserm UMRS 938, Université Pierre et Marie Curie Paris 6, Paris, France

**Sir,**

We read with great interest the letter of Peres *et al* (2012) regarding our article ‘Contribution of pelvic and para-aortic lymphadenectomy with sentinel node (SN) biopsy in patients with IB2–IIB cervical cancer’.

Nevertheless, we would like to discuss their comments.

First, effectively, the goal of SN biopsy in locally advanced cervical cancer (LACC) is different from that proposed in early stages. In early stages, the role of the SN biopsy is to avoid systematic lymphadenectomy, as the risk of lymph node involvement is <20% while exposing patients to the risk of lymphocele and lymphoedema. Previous studies (Altgassen *et al*, 2008; Lécuru *et al*, 2011) have demonstrated the relevance of the SN biopsy to detect not only lymph node macrometastases but also micrometastases that have a negative impact on the recurrence rate. Indeed, Juretzka *et al* (2004) and Marchiole *et al* (2005) have underlined the risk of recurrence associated to micrometastasis justifiying and adjuvant therapy. From pathological viewpoint, the detection of pelvic micrometastasis by ultrastaging can contribute to discuss the concept of skip metastases that are usually reported in 1–2% of cases. In fact, these metastases are probably associated with non-detected pelvic metastasis by routine histology.

Second, we agree that there is a low detection rate per hemipelvis and per patient in our study. Lécuru *et al* (2011) in a prospective multicentre study have reported a bilateral detection in only 76.5% of the patients with early-stage disease. Our rate is effectively lower as expected link to tumour volumes and obstruction of lymphatic channel by tumour cells. However, the advantage of this procedure is to detect some patients with pelvic involvement requiring in addition to classic concomitant radiochemotherapy (CRC), an adjuvant chemotherapy. Indeed, a recent meta-analysis has underlined the potential benefit of such a strategy in patients with high risk of recurrence (Chemoradiotherapy for Cervical Cancer Meta-analysis Collaboration (CCCMAC), 2010).

Third, another potential interest of the SN biopsy is to adapt adjuvant therapy. Indeed, it is well known that the rate of para-aortic lymph node involvement, even in patients with LACC, is about 25% of cases. Therefore, the prognosis for the remaining patients depends on two further parameters: first, an incomplete response to CRC of primary tumour addressing the issue on completion of surgery; and second, the presence of pelvic metastasis observed in 50% of our population. In this specific setting, some authors have underlined the risk of pelvic side recurrence related to lymph node metastases not controlled by CRC, raising the issue on the potential therapeutic effect of pelvic lymphadenectomy for LACC (Delpech *et al*, 2007). Moreover, as mentioned in our article, only two patients had isolated para-aortic metastasis, suggesting that knowledge on pelvic lymph node status seems crucial.

In conclusion, although the relevance of the SN biopsy in LACC could be discussed, as an alternative of systematic lymphadenectomy, our results underlined the potential contribution of the SN biopsy with ultrastaging to better adapt both surgical management and adjuvant therapy to improved survival.
